# Health systems and the SDGs: lessons from a joint HIV and sexual and reproductive health and rights response

**DOI:** 10.1093/heapol/czx052

**Published:** 2017-11-24

**Authors:** Charlotte E Warren, Jonathan Hopkins, Manjulaa Narasimhan, Lynn Collins, Ian Askew, Susannah H Mayhew

**Affiliations:** 1 Population Council, Suite 280, 4301 Connecticut Avenue NW, Washington, DC 20008, USA; 2 International Planned Parenthood Foundation, 4 Newhams Row, London SE1 3UZ, UK; 3 World Health Organization, 20 Avenue Appia, 1211 Geneva 27, Switzerland; 4 UNFPA, 605 Third Avenue, New York, NY 10158, USA and; 5 Faculty of Public Health & Policy, London School of Hygiene and Tropical Medicine, 15-17 Tavistock Place, London WC1H 9SH, UK

## Introduction

This commentary article, in the *Integration for stronger health systems: Lessons from sexual and reproductive health integration* Supplement, discusses the lessons from research on integrated sexual and reproductive health (SRH) and HIV programming over the last decade and maps out strategies for a health systems strengthening approach to achieving the sustainable development goals (SDGs).

The experiences of decades of integration of sexual and reproductive health and rights (SRHR) and HIV responses can provide insights on how to progress towards the SDGs (UN 2015). Integration makes use of different service entry points, reduces structural silos, enables efficiencies and builds a broader cross cutting approach, to deliver comprehensive care for clients with multiple health needs (Johnstone et al. 2013; WHO 2015a). In 2015, the SDGs were launched to shape the next 15-year development agenda, and complete the unfinished agenda of some of the Millennium Development Goals (UN 2015). SDG Goal 3—good health and wellbeing—focuses most explicitly on health and covers two major targets relevant to this article; SDG 3.3—end the epidemics of AIDS, tuberculosis, malaria and neglected tropical diseases and combat hepatitis, water-borne diseases and other communicable diseases; and SDG 3.7—ensure universal access to sexual and reproductive health-care services, including for family planning (FP), information and education, and the integration of reproductive health into national strategies and programmes (UN 2015). A key lesson from the Millennium Development Goals is that the new health goal and related targets cannot be achieved without (1) strengthening health systems broadly, and (2) linking the health sector with other sectors that address the structural determinants of health.

This article builds on the growing body of evidence of the benefits of SRH and HIV service integration, to describe lessons learned and evidence informed applications from the evidence that can be applied to the SDGs. SRH services are seen as an example of a broader platform that is not disease specific, but focuses more on a person-centric, holistic approach to health and well-being that could contribute to the wider discourse on the opportunities and known challenges for integrating health services. The lessons learned both within and beyond the health sector to the wider agenda of linking policies and programs on SRHR and HIV can inform future strategies towards a multi-sectoral developmental approach to attain the SDGs.

### Integration or linkages?

One fundamental challenge has been developing—and agreeing on—a definition of “integration”. Integration “*can be understood as joining operational programmes to ensure effective outcomes through many modalities; multi-tasked providers, referral, ‘one-stop shop’, services under one roof*…” (UNFPA, WHO & IPPF 2017). An integrated health system is potentially more cost effective and helps maximize the use of limited health resources and provide a more comprehensive package of health care for the users (Sweeney et al. 2012; Lassi et al. 2013; Obure et al. 2015). At its simplest it is “*…combining different kinds of services to maximize outcomes*” (UNAIDS 2011). Integration can be *bi-directional*, with SRH services integrated into HIV services and vice versa; and can be ‘*subdivided’* at facility level in terms of four dimensions: *structural integration* (services availability, resources, location); *functional integration* (the range of services each client receives per visit); *temporal integration* (range of services accessed daily/days per week) and *provider integration* (services provided per staff per day) (Mayhew et al. 2015).

It is important to note that health *service* integration alone is inadequate for achieving integrated care and improving health outcomes (Hope et al. 2014). Integrating SRH and HIV services contributes to, but also requires, strengthening health systems and the wider agenda of linking policies and programs demands a multi-sectoral approach. A health systems perspective is required to build and exploit the synergies both within and across delivery of services and to connect with other sectors. A multi-sectoral perspective, for example the health sector working with ministries of Education, Gender, Social Services and Youth and Sports, achieves a more holistic response to sexual and reproductive health (Danida 2014; Mayhew et al. 2015). This broader approach has been termed ‘*linkages*’ and refers to the synergies in the enabling environment (laws, policies, funding), health systems (planning and coordination, capacity-building, commodities, monitoring and evaluation), and includes integrated service delivery of SRH and HIV (IPPF et al. 2009, WHO 2015b).

## A history of SRHR and HIV integration

The debate on whether and how to integrate related but conventionally separate packages of health services has a long history, originating from the Alma Ata Declaration on Primary Health Care (PHC) in 1978 to provide ‘Health for All by the Year 2000’, and founded on principles of equity, inter-sectoral collaboration and community participation (WHO 1978). However, commitment to this broad approach for health service delivery receded when donors started to support countries to provide "*selective PHC*"—setting the precedent for decades of vertical ‘siloed’ programming and donor funding. The selective approach proposed targeting the most severe public health problems to maximize improvement of health in low and middle income countries which became focused on four vertical programs: infant growth monitoring, oral rehydration therapy, breastfeeding and immunization. FP, female education and food supplementation were added later (Magnussen et al. 2004). The focus on hospital-based, disease-oriented and autonomous and primarily curative health services models weakened the ability of health systems to provide universal, equitable, high-quality and financially sustainable care (WHO 2015).

During the 1980s, women’s rights movements argued for a more holistic approach to sexual and reproductive health including the integration of STI services. In 1994, the International Conference on Population & Development (ICPD) articulated a commitment to ensuring women’s and girls’ rights to a comprehensive package of SRH services, and promoted a concept of women’s health beyond childbearing that encompassed notions of empowerment, gender equality, protection of human rights and a life-cycle approach to SRH, including maternal health. Post ICPD, the escalation of the global HIV epidemic focused attention on the need to rapidly expand access to HIV screening and later to treatment and then to integrate HIV prevention services within multiple SRH (including maternal health) services—in particular FP and antenatal and postnatal care (Grosskurth 1995; Mayhew 2000; Shelton 1999; Foreit et al. 2002). The rationale and benefits for integrating HIV prevention and care with SRH services have been articulated in a number of reviews, studies and policy documents (e.g. Mayhew 1996; Askew and Berer 2003; WHO et al. 2005; Church and Mayhew 2009; Kennedy et al. 2010). However, there was no consensus on which services should be offered together and which should be maintained as standalone services and in specific contexts (Dehne et al. 2000), which depended on the extent of the HIV epidemic, strength of any particular health system and national priorities.

**Figure 1.**
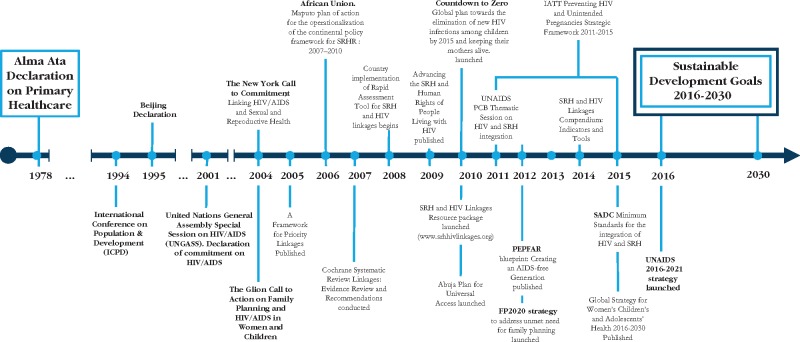
Key dates in the field of HIV and SRHR

Moreover, the impact of greatly increased and targeted HIV financing at country level, coupled with reductions in SRH funds, risked undermining efforts at system strengthening and effective service integration and policy linkages (Druce et al. 2006; Spaulding et al. 2009; WHO UNFPA IPPF & UNAIDs 2005; WHO, UNFPA, IPPF, UNAIDS & UCSF 2009). Following the ‘Glion Call to Action on Family Planning and HIV/AIDS in Women and Children’ and the ‘New York Call to Commitment: linking HIV/AIDS and SRHR’ (UNFPA 2004) the Inter-Agency Working Group (IAWG) on SRHR and HIV linkages was established, co-convened by WHO and UNFPA, bringing together UNAIDS, IPPF, UNDP, donors and development partners. The Working Group is committed to intensifying SRH and HIV service integration and the broader linkages through advocacy, policy and programmatic efforts. A framework was proposed that outlines a set of key policy and programme actions to strengthen linkages between SRHR and HIV/AIDS in 2005 (see Figure 1). The IAWG also developed tools for countries to assess the extent to which their policies and health systems are integrated as a first step to providing or strengthening integrated services (IAWG on SRHR and HIV Linkages 2010). Since 2010 there has been an increase in global strategies, plans and tools to strengthen the case for integrating SRH and HIV health services as well as SRHR and HIV linkages broadly beyond the health facility. Figure 1 outlines the timeline of key global policies and statements from the Alma Ata declaration for Primary Health Care in 1978 to date.

From the mid-2000s, a number of large research projects began that focused specifically on integrated SRH and HIV services. These included the FACES project led by University California, San Francisco (UCSF) and the Kenya Medical Research Institute (KEMRI) in southwestern Kenya, and a range of studies conducted by FHI360, both of which focused on integration of FP services into HIV services (Grossman et al 2013; Wilcher et al. 2013). The Integra Initiative, a multi-country study implemented by IPPF, the London School for Hygiene and Tropical Medicine, and Population Council, focused on SRH services as the entry point for integrating HIV services. Integra evaluated the potential benefits, costs and efficiencies of different integration models in Kenya, Malawi and Swaziland (Warren et al. 2012). The models included HIV services integrated into FP, HIV services integrated into postnatal care in 32 public health facilities, and HIV services integrated into broader SRH services in IPPF clinics. An SRHR and HIV linkages project for seven countries in Southern Africa funded by EU/NORAD/SIDA and implemented by UNFPA and UNAIDS was initiated in 2011 and focused its work on linking SRHR-HIV at policy and system level and modelling integrated SRH and HIV at service delivery level (UNFPA 2015).

The integration of HIV with other health services is seen as an important mechanism to overcome verticalization and strengthen health systems (Coovadia and Bland 2008; Church et al. 2015). However, integrating two obviously related fields of healthcare—SRH and HIV—has proved challenging, when the policy rhetoric of integration is often at odds with the frequently compartmentalized service delivery reality in lower-income settings. In addition Ministries of Health (MOH), national AIDS coordinating bodies, and donors have typically ‘siloed’ policies, programmes and financing (Hope et al. 2014). Hopkins and Collins (in this Supplement) reviewed HIV and SRHR national strategies in over 60 countries between 2008 and 2015 to assess whether HIV strategies mention SRHR and *vice versa*. Even where clear rhetoric for linking HIV and SRHR exists at the policy level—especially since the New York Call to Commitment and Glion Call to Action in 2004—there was limited evidence on whether policy guideline development or service integration had actually taken place.

In the last few years, implementation research on how best to integrate these two closely related services has informed many international, regional and national health policies and programmes so that they support some degree of integration at the service delivery level. The growing evidence of integrated services is encouraging (Church and Mayhew 2009; Spaulding et al. 2009; Kennedy et al. 2010; Global Fund 2011; Obure 2015; Johnstone et al. 2013; Hope et al. 2014; WHO 2015a), but it highlights the need to resolve health systems obstacles to enable scale up of integrated service provision (Wilcher et al. 2013; Mayhew et al. 2016).

## What have we learned? Strengthening health systems and integrated health service delivery

From a systems perspective, integration requires attention to structural elements of joint planning, financing and collaboration between departments within the health system that do not necessarily work together, for example, for the procurement of supplies, management of human resources, development of new clinical guidelines and training of health care providers to provide integrated services (WHO 2007; WHO, USAID, FHI 2009). The specific package of integrated services for a country is based on both the context of a particular population’s health needs and a consideration of the structure of the health system and available resources (WHO 2007).

More than this, however, systems must support the people within them. Evidence from the *Integra Initiative* (Mayhew et al in this supplement) suggests a people-centred approach to integration can successfully overcome some structural deficiencies. Specifically, when health providers have agency to make decisions, and are able to work together effectively in teams, they are better able to manage the diverse skills needed when providing integrated services and can cross-refer clients to call on others to deliver a service if they are not able to themselves. Consequently they can provide a more responsive package of care to a client (or client-centred care) during a single visit to the health facility. However, management systems need to be able to support providers to make flexible decisions and facilitate better coordination and communication across clinics within facilities (Colombini et al. 2016b).

Importantly, an analysis of the effects of integration indicates that it has the potential to improve quality of care (Mutemwa et al in this supplement), and that repeated access to integrated services can improve clients’ health outcomes, including increasing use of both FP and HIV testing and counseling (Kimani 2015, Mutemwa et al 2016 and Church et al 2017). *Integra* also showed the importance of sensitizing providers to the potential risks for partner violence following disclosure of HIV testing and ensuring that the woman’s decision to disclose is fully informed and voluntary is an important element of counseling (Colombini et al. 2016a). Integration also has the potential to facilitate efficiency gains in some models by optimizing workload when integrating HIV into FP or postnatal models of care (Sweeney et al. 2012).

In southwestern Kenya, where FP was integrated into HIV care and treatment, researchers found a higher proportion of women using effective contraception than women referred to an FP clinic in the same facility (Grossman et al. 2013). Integration was both cost efficient (cost per additional use of more effective FP) and cost effective (cost per pregnancy diverted) and acceptability was high among women and providers, demonstrating a promising approach to increase use of more effective FP among women living with HIV (Shade et al. 2013; Patel et al. 2014). Men, however, preferred to receive FP information in HIV care and treatment sessions (Steinfield et al. 2013). A review of best practices on integration of FP into HIV programs describes how the evidence base is growing, with an increasing number of guidance documents and tools that are available to support integrated programming. However, integration will only advance and be sustained if system constraints are addressed and linkages at higher levels within and beyond the health system are strengthened (Wilcher et al. 2013).

## What have we learned? SRHR and HIV linkages

Research on linkages (as distinct from service integration) between SRHR and HIV policies suggests that better health outcomes can be achieved when health services, systems and the enabling environment (including supportive policies) take into account social issues and include respect for clients health and human rights; are able to connect multiple service-components through effective linkages between health professionals within the system and who are motivated and enabled to make connections beyond their usual responsibilities, to decrease barriers to access (UNAIDS 2010; Mayhew et al. 2016; UNFPA, WHO & IPPF 2017). Research on country experiences of linking SRH and HIV programmes has also shown that both strong political will and governance are necessary ( Lusti-Narasimhan et al. 2014; Mayhew et al. 2015; Waage et al. 2015; Hopkins and Collins 2017 in this supplement).

Promoting links between SRH and HIV programmes has also underlined the importance of consolidated involvement by civil society organizations (CSOs) in advocating for integrated services and cross-sector synergies for accountability of governments pledging to act. The actions of CSOs are critical in ensuring that structural factors in the enabling environment (such as realization of rights) are not neglected. Achieving universal access to quality healthcare will not be possible without addressing the structural determinants of health and are part of the reason why a SRHR and HIV linkages approach is so integral to a broader human rights framework (WHO 2015a; UNFPA, WHO & IPPF 2017). SRHR and HIV civil society movements recognize that the structural determinants that drive HIV and poor SRHR status go well beyond the health sector and cannot be overlooked (UNAIDS 2010).

In particular stigma and discrimination and repressive laws and policies hinder access to many SRHR and HIV services for young people and key populations such as men who have sex with men, sex workers, people who inject drugs and transgender people (UNAIDS 2010, 2011). There are particularly pertinent examples from gender-based violence research. Any comprehensive response to gender based violence must involve the justice sector (e.g. to agree protocols acceptable to police for pursuing prosecution); referral networks to specialist post-rape services, and challenge assumptions about women’s empowerment including addressing economic empowerment of marginalized women could reduce violence against women (WHO 2005; Morrison et al. 2007; Rocca et al. 2009; Abramsky et al. 2014).

### Applications for SRHR and HIV linkages approach to the SDGs

Integration of SRH and HIV interventions or programmes within the broader linkages approach can provide useful insights for the implementation of the SDGs: recognising and mapping interconnectivity at various levels, including within and beyond the health sector; the critical role of political will and governance; and the importance of civil society for ensuring accountability.

The SDGs offer both opportunities and challenges for the SRHR and HIV linkages agenda. A key opportunity is the unified health goal to ‘Ensure healthy lives and promote well-being for all at all ages’ (SDG 3) which necessitates a broader approach. This focus on ensuring health and well-being as well as protecting against death and morbidity from specific diseases will require—in theory—better linked policies and programming, particularly if universal health coverage is to be achieved. In the context of the SDGs, health must be seen not just as a multi-component goal within a single sector, but as a multi-sector product—as indeed has always been the case, but seldom operationalized (EWEC 2015; WHO 2015a; WHO 2015b). The challenge is how to achieve meaningful progress on health outcomes in this complexity. Linking strategies beyond Health Goal 3 to ensure healthy lives and promote well-being for all at all ages, such as the SDG target 3.7—achieving universal access to sexual and reproductive health and the Gender Goal 5 to achieve gender equality and empower all women and girls (SDG 5.6)—is therefore critical.

Recognizing the interconnectivity of the SDGs, and specifically the linkages between SDG Health Goal 3.7 and SDG Gender Goal 5.6, UNAIDS and UNFPA have mapped HIV and SRHR programme elements across the SDGs. UNAIDS highlight how HIV impacts progress towards select SDGs, and identifies opportunities for cross-sectoral collaboration towards shared goals for 2030 (UNAIDS 2016). UNFPA has mapped the SDGs from an SRHR perspective which focus on the full scope of the linkages agenda from both human and reproductive rights perspectives, including addressing child marriage, gender-based violence, stigma and discrimination, comprehensive sexuality education, empowerment of women, and the rights of young people (UNFPA 2016). The SDGs refer several times to the term “human right(s)” (rights to development, self-determination, an adequate standard of living, food, water and sanitation, good governance, and the rule of law), though does not specifically mention that health is a human right (WHO 2015a).

The concept of linkages within and across the enabling environment, health systems and service delivery, has considerable resonance for the current dialogue on how health can benefit from—rather than be lost within—the wide-ranging multi-sectoral SDG platform. By taking a more integrated approach to provision of health services, the SDGs present an opportunity to advance, collaborate and capitalize on the synergies as well as holding global and country policy makers to account as called for in the Accountability Framework for the Global Strategy for Women’s, Children’s and Adolescents’ health (EWEC 2015). The SDGs provide an opportunity to rethink approaches to equitable health coverage, integrate marginalised populations, and enshrine a stronger focus on human rights.

## Looking forward to 2030: recommendations for creating a linked SDG response

It is clear that an integrated approach is gaining traction as an important way forward, but this will not occur without concerted effort and changes to the ways in which the historically vertical programming of HIV and SRH services has been designed, funded, implemented and monitored. Universal access to health and well-being has been proposed as underpinning all other SDGs (WHO 2015.6) and so there is clearly an opportunity to refocus efforts on a more sustainable approach through system-wide support that also enshrines protection of rights and cross-sector collaboration to achieve this. Lessons from the rich body of research on integrating SRH and HIV health services highlight the need for health systems to make connections within and beyond the health sector. This can be done by supporting the health workforce within the system to link with others (which will be heavily shaped by the governance frameworks in place); to promote a more supportive enabling environment (of policies, structures and social understanding); and to establish clear structures in holding governments and government agencies accountable for action on linkages (Waage et al. 2015).

Scale-up of programmes and strengthening of existing systems create new opportunities for program and service integration. Concerted efforts at global and country level are required to ensure that the numerous overlapping strategies for reaching UHC, including SDG targets 3.3, 3.7 and 5.6, Global Strategy for Women’s, Children’s and Adolescents’ Health (2016–2030), FP2020, Creating an AIDS Free Generation (2012) and others learn lessons from and build upon previous efforts.

Some recommendations can be identified which require explicit policy direction. There is a well-established rationale for integrating SRH and HIV health services in the broader context of SDGs, especially in LMICs. For policy makers, the progressive realization to the right to health and developing enabling environments to support the structural linkages for planning and service-delivery across sectors is critical and requires political will and strong leadership (UNFPA, WHO & IPPF 2017). Policy makers and programme managers need to be supported and enabled to put into practice key action-areas through a people-centred cross-sectoral approach. The action of duty bearers (governments, donors, CSOs, health system actors) is important in ensuring that structural factors in the enabling environment, and especially realization of rights, are not neglected. The challenge is whether policy and management levels of the system do indeed operationalize the elements they commit to. Researchers have an obligation to rights holders (client users of the services) to systematically map and analyse the connections, and the impacts of those connections, between health systems and the SDGs if the health and wellbeing goal is to be realized as a fundamental component underpinning the SDGs.
